# 

**Published:** 1888-12-01

**Authors:** 


					W u PERMANENTLY ENLARGED TO 32 PAGES.
? PRICE ONE PENNY. w.w a .
^ 114, vol V.-
December 1st, 1888.
?r? rer Annum, 6s. 6d., post free.
Monthly Parts. 6d.; post free, 7id.
IttegtoJered at the General Poit Office ?^
as a Netrgpaper.] '<?? v Ditto per Aax. 7s. 61. post free.

				

